# Evaluation of diode laser application on chemical analysis and surface microhardness of white spots enamel lesions with two remineralizing agents

**DOI:** 10.4317/jced.56490

**Published:** 2020-03-01

**Authors:** Lamiaa-Mahmoud Moharam, Doaa-Mohamed Sadony, Shaymaa-Mohamed Nagi

**Affiliations:** 1 Restorative and Dental Materials department, National Research Centre, Giza, Egypt

## Abstract

**Background:**

To investigate the effect of diode laser application and two commercial remineralizing agents on the remineralization and surface microhardness of white spot enamel lesions.

**Material and Methods:**

Sixty specimens were prepared then equally divided into six groups (n=10/group), according to the diode laser and the two commercial remineralizing agents applied to demineralized enamel surfaces (APF gel and sodium fluoride NaF mousse) with or without diode Laser application as follows; Group A; control, Group B; diode Laser application, Group C; APF gel application, Group D; NaF mousse application, Group E; APF gel application + diode Laser, Group F; NaF mousse application+ diode Laser. Then the teeth were investigated for their Ca, P & F ions content and surface microhardness. One-way ANOVA followed by Tukey’s (HSD) post hoc test were used for statistical analysis.

**Results:**

Ca ion wt% showed no statistically significant difference between tested groups, with the highest mean value recorded with Group C. P ion wt%, showed a statistically significant difference between Groups A and C, and the highest mean value was recorded for Group A. The highest F ion wt% was recorded for Group C, while the lowest was recorded for both A and B groups. The highest significant microhardness mean values was recorded for Group E, while the lowest was recorded for Group A.

**Conclusions:**

Diode Laser treatment of the demineralized enamel surface had a positive influence on the chemistry and surface microhardness and it may represent a promising adjunct for enamel surface remineralization.

** Key words:**Diode Laser application, chemical analysis, surface microhardness, remineralizing agents, white spot lesions.

## Introduction

Modern dentistry has introduced “prevention” of dental caries as one of its main goals. Another main goal is “remineralization” of the demineralized enamel and dentin tooth structures rather than the conventional “drill and fill” dental caries treatment. Such goals can be successfully achieved through delivering fluoride, calcium and phosphorus ions to the tooth surface (1), which can be found in the form of mouthwashes, toothpastes, pit-and-fissure sealants, gels and among many others.

Generally, lasers can represent a new treatment modality for Fighting dental caries. Recent researches in the earlier few years conveyed the merits of using infrared radiation of lasers on enamel surface specifically, whether used on their own (2) or along with the application of different remineralizing agents such as fluorides (3), that has been proofed to increase enamel resistance to acid attacks or enhance the uptake of fluoride, so that the enamel will be more resistant to dental caries (4) and different acidic attacks.

The consequences of application of diode laser with a λ of 809–960 nm on the enamel surface were only investigated in a limited number of studies. The hydroxyapatite of the dental structure absorbs low levels of this λ, while the rest is being transferred as heat on the enamel surface and its nearby structures (5).

However, such elevated temperature of the enamel may correspondingly yield some serious alterations in the structure and ultrastructure of the enamel, which will lead to a decrease in the enamel-acid dissolution tendency. Such variations may embrace the destruction of its organic matrix, carbonate and water loss, besides the development of an acid-resistant hydroxyapatite layers (6).

It was formerly stated that the combination of diode laser with sodium fluoride application was found to be efficient in elevating the levels of fluoride uptake by dental enamel (7). Nevertheless, other researches revealed a noteworthy reduction in the enamel-acid solubility and the hindrance of carious lesions development *in vitro* (8).

Therefore, the aim beyond the current study was to evaluate the efficacy of the application of diode laser on chemical analysis and surface microhardness of white spot enamel lesions with or without the application of fluoride remineralizing agents.

The null hypotheses investigated was that the different tested remineralization protocols applied to white spot lesions have no effect on enamel chemistry and surface microhardness.

## Material and Methods

-Selected Materials:

Two commercial remineralizing agents were tested in this study; Acidulated Phosphate Fluoride (APF gel) (Alpha-PRO®APF) and Sodium fluoride (WHITE smile Mousse). The materials brand name, description, composition and their manufacturers are listed in Table 1.

**Table 1:**
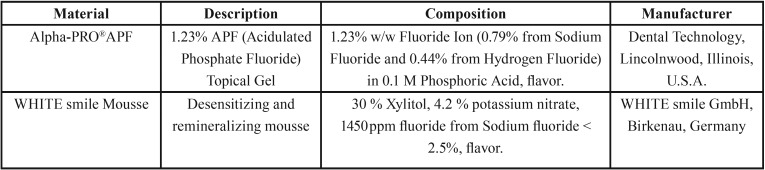
Materials, description, composition and manufacturer.

-Study design and specimens grouping

Sixty extracted anterior bovine teeth were equally divided into six groups (n=10/group), according to the remineralization protocols used.

Group A; White spot enamel lesions received no treatment (control).

Group B; Diode Laser application.

Group C; APF gel application.

Group D; NaF mousse application.

Group E; APF gel application+ Diode Laser.

Group F; NaF mousse application + Diode Laser.

Sample size calculation was done using R statistical package, version 2.15.2 (26-10-2012). Copyright (C) 2012 - The R Foundation for Statistical Computing. In a one-way ANOVA study, results showed that a total sample size of 10 samples will be adequate to detect a mean difference between study groups with a power of 80% and a two-sided significance level of 5%.

-Teeth selection and preparation

A total of 60 bovine anterior teeth were selected for this study. Teeth were scraped with hand scaler and washed under running tap water to remove any residual tissues and debris. The roots of the cleaned teeth were cut with a double side-cutting low-speed disc at the level of the enamel-cementum junction.

The pulp tissues were removed using barded broaches and the pulp chambers were closed using pink wax. The enamel surfaces were ground under wet conditions with 800-1000 grit silicon carbide abrasive paper to obtain standardized flat surfaces of enamel.

-Formation of enamel white spot lesions

Acid resistant varnish (Nail polish, Barielle, NY, USA) was painted all over the labial surface of the crowns except for a single window of 3 mm X 3 mm at the cervical 1/3 of the crown (9). After complete setting of the nail varnish all specimens were suspended in 20 ml of acidified buffered demineralizing solution for 48 h at 37 °C. The demineralized solution contained 2.2 mmol/L CaCl2, 2.2 mmol/L NaH2PO4 and 50 mmol/L acetic acid adjusted to pH 4.5 with NaOH (10).

Application of the different remineralization protocols:

Specimens were rinsed thoroughly using deionized water and dried gently using clean filter papers. The two remineralizing agents Alpha-PRO®APF gel and WHITE smile (NaF) mousse were applied to the marked windows on the labial surface of the assigned specimens of the tested groups using cotton tips. The applied layers were 1-2 mm thick and were left undisturbed in place (11) for 60s according to their manufacturers’ instructions, either alone (groups C and D) or followed by immediate application of the diode laser (groups E and F). Specimens were then washed thoroughly using deionized water for one minute to remove any visible remnants of the gel.

-Diode Laser application

A commercially available diode laser (Gallium-Aluminium-Arsenide (Ga AlAs) diode laser, Siro-Laser Advance class III b, SIRONA Dental Company, Germany) of 970-nm wavelength and a power of 2W was used in a continuous mode to the assigned prepared teeth surfaces for 15s (12,13). It was attached to a 220-μm optic conductor fibre as a transmission element.

The laser beam was applied to the marked windows on the cervical portion of labial surfaces of prepared teeth crowns either alone (group B) or applied immediately after the application of the two remineralizing agents (groups E and F).

The tip of the optic fibre was placed at a non-contact mode with a standard distance of 2-mm (14), using putty moulds to assure an equal distance between the tip of the optic fibre and the enamel (15).

The optic fibre was moving uniformly and longitudinally over the marked window (4) by the same operator for all the specimens. After lasing, the specimens were rinsed thoroughly using deionized water for one minute to remove any visible remnants of the remineralizing agents.

Then the specimens were stored in deionized water, in tightly sealed polyethylene test tubes at 37ºC for 24h before the tests were conducted.

-Chemical analysis assessment testing

The prepared specimens were assessed for remineralization through evaluation of Calcium, Phosphorus and Fluoride ions content using environmental Energy Dispersive X-ray Analysis (EDX, Model Quanta 250, FEI company, Netherlands).

The specimens were placed on aluminium stubs inside a chamber with their labial enamel surfaces facing upwards. Using compressed air, the water covering the surface was gently removed. Then the Ca, *P* and F ions contents were determined in wt%.

-Surface microhardness testing

The prepared crowns specimens were centralized and set horizontally in auto-polymerized acrylic resin blocks with their treated labial surfaces facing upward using rounded sectional Teflon molds of 4 cm diameter.

The acrylic resin was left to set for one hour, then the enamel surface microhardness was assessed using Digital Vickers microhardness tester (NEXUS 4000TM, INNOVTEST, model number 4503, The Netherlands), using 20X magnification, a load of 200 g and a dwell time of 10 s (16).

Three randomized indentations were made on the marked window of the treated labial enamel surfaces of the prepared specimens. Surface microhardness calculations were done using computer software (Hardness-Course Vickers/ Brinell/ Rockwell copy right IBS 2012 version 10.4.4).

-Statistical analysis

Mean and standard deviation values were calculated for each group. Data showed parametric distribution. One-way ANOVA was used to show the difference between the tested groups followed by Tukey’s (HSD) post hoc test for pairwise comparison. A value of α = 0.05 was used as the level for significance. Statistical analysis was performed with IBM SPSS Statistics for Windows, Version 23.0. Armonk, NY, USA.

## Results

Data in Table 2 shows the results of one-way ANOVA analysis for the chemical analysis assessment of the white spot enamel lesions using two remineralizing agents with or without diode laser application. For Calcium enamel content (wt%), there was no statistically significant difference between the tested groups at *p*=0.809. The highest mean values were recorded for group E (68.44±3.14) while the lowest was recorded for group A (66.72±0.61). For Phosphate enamel content (wt%), there was a statistical significance between group A on one hand and E, F on the other hand, while there was no significant difference between groups B, C, D on one hand and group F on the other hand. There was no statistically significant difference between groups B, C, D and F at *p*=0.01. The highest mean value was recorded for group A (33.01±0.62) while the lowest was for group E (28.59±2.46).

For Fluoride enamel content (wt%), there was a significant difference between groups F, E on one hand and between A, B, C, D on the other hand at *p*=0.001. The highest mean wt% value was recorded for group E (2.97±0.72), while the lowest was for both group A and B (0.27±0.02).

**Table 2:**
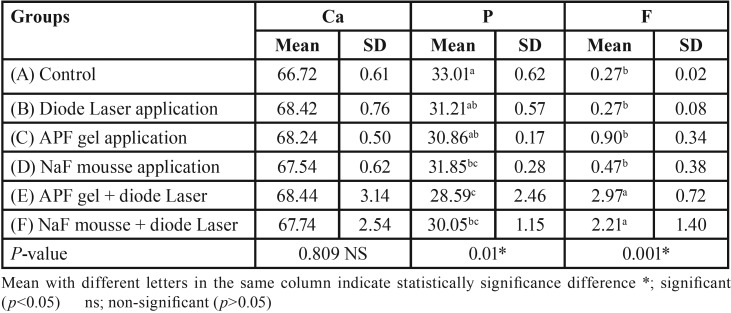
One-way ANOVA test for chemical assessment of the different tested groups.

Data in Table 3 shows the mean, standard deviation (SD) values of the effect of the two remineralizing agents with and without diode laser application on the surface microhardness of the white spot enamel lesions.

**Table 3:**
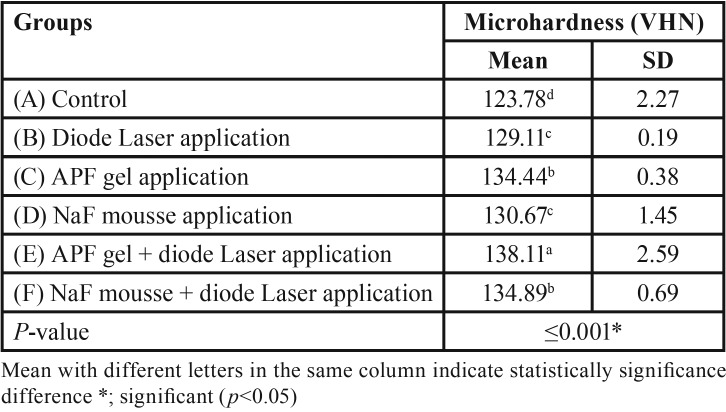
One-way ANOVA test for surface microhardness of different tested groups.

There was a statistically significant difference between the tested groups at *p*≤0.001. However, there was no significant difference between groups C, F and between groups B, D. The highest mean microhardness value was recorded for group E (138.11±2.59), while the lowest was recorded for group A (123.78±2.27).

## Discussion

In the past few years there has been a widespread in the use of diode lasers in different fields of dentistry. It was reported that red and near-infrared low power lasers represent an alternate tactic in caries prevention. Some studies proposed that their application with or without topical fluoride treatment, can increase the resistance of teeth against dental caries (17).

Some researchers stated that laser light must be efficiently absorbed and transformed into heat without injury to underlying tissues. This way Laser light can successfully modify the solubility and composition of dental hard tissues (18).

In this study, the treatment protocol comprised diode laser irradiation and fluoride application combination. Although, the precise mechanism of enamel fluoride uptake is not yet clear. However, two mechanisms have been suggested: first, is the effect of heat generated by laser to increase the fluoride uptake by the enamel (19), and second, is the micro-cracks created on the enamel surface due to the laser treatment which retain fluoride inside (20).

In this study, the results of the chemical analysis of the tested specimens using the environmental EDX (Table 2) revealed that calcium enamel content (wt%) was maintained and even showed an insignificant increase through the control and experimental groups (with or without diode laser irradiation as well as with and without fluoride application).

These might be due to the demineralization period of the specimens in the current study which was limited to 48 h, that might not be enough to remove a significant level of the calcium enamel content. These findings agreed with the results of de Sant’anna *et al.* (18), who reported in their results that the calcium enamel content was maintained between the different tested groups using diode laser irradiation and photo-absorbing fluoride cream.

Such results could be further explained by Simmer and Fincham (21), they reported that the calcium activity level will be raised to the 10th power in the solubility product equation due to the presence of 10 ions of calcium per unit in the calcium hydroxyapatite, regarding that the enamel solubility product which directly affects the resistance of enamel during the caries process or different acidic challenges.

Consequently, dental enamel solubility product is further affected by calcium concentration changes more than by changes in other parameters.

Furthermore, phosphorus enamel content (wt%) was maintained with a slight significant decrease through all tested groups.

These results agreed with those of Antunes *et al.* (22), they owed the reduction in the phosphorus content after laser irradiation to a volatilization caused by heat generation. Nevertheless, they used high power Nd:YAG lasers, while low power diode laser was used in the current study.

This results inconsistency can be elucidated by our use of a low power diode laser in continuous mode for a short duration, which might have favored thermal-relaxation of the enamel and lowered the incidence of disagreeable effects.

On the other hand, fluoride enamel content (wt%) was higher in groups treated with both fluoride agents and irradiated with diode laser than in the control group, diode laser irradiated group and unirradiated fluoride treated group.

This might be attributed to the efficiency of the diode laser to decrease the acid-solubility of enamel surface. Moreover, using laser with a fluoride agent could increase the fluoride uptake by the treated enamel. These results were in accordance with Gonzalez-Rodriguez *et al.* (4) and Villalba-Moreno *et al.* (7), whose results showed a significant increase in fluoride uptake in the enamel surface of the tested specimens following laser treatment.

However, their readings were generally higher than the results of the current study, which might be due to using different testing devices and techniques to measure the fluoride concentrations as well as using different diode lasers of higher powers and outputs.

Regardless of the application of diode laser, using APF gel topical fluoride application showed higher fluoride enamel content (wt%) values than those of NaF mousse, which might be owed to the higher concentration of the fluoride in APF gel (12,300 ppm of fluoride compared to 1450  ppm fluoride of the NaF mousse) according to their manufacturers’ materials safety data sheets. Moreover, the gel consistency of the APF gel might have allowed more intimate contact between the gel and the enamel, leading to elevated fluoride uptake levels by the enamel surface.

The surface layer of the tooth plays a significant role in the progression of dental caries. Hence, the evaluation of changes in this area is of great importance.

Vickers microhardness test represents a proper procedure for such purpose, and it is convenient for materials with fine micro-structure and non-homogenous like dental enamel. Micro-hardness test offers a rather simple, rapid and non-destructive method in demineralization and remineralization studies of the hard tooth structures (10).

Moreover, a significant correlation was reported between the surface microhardness of enamel and mineral loss in carious lesions (23).

Vickers micro-hardness test results of the current study revealed a significant increase in both laser-irradiated fluoride-treated groups over the control and other experimental groups. This might be attributed to the numerous physico-chemical alternations, which have been proposed to rise during laser-irradiated fluoride treatment of the dental hard structures that comprise calcium-fluoride deposition (24), formation of micro-spaces in the dental hard tissue (20), formation of tri-calcium phosphate (25), and hydroxyapatite crystals transformation into fluorapatite (26).

These findings agreed with those of Vlacic *et al.* (27). They reported the success of laser-activated fluoride treatment using various laser wavelengths, in increasing the micro-hardness of demineralized enamel.

It was reported by Fox *et al.* (28) that the reduction in the dissolution rate of enamel surface is greatly affected by laser application. This was also supported by Nammour *et al* (29), who showed that *in vivo* application of argon laser allowed more fluoride retention compared to un-lased enamel, due to the obvious structural changes in the lased enamel that produced a reservoir.

Moreover, the results of this study showed that using APF gel with or without laser application, showed higher microhardness values than those of NaF mousse, which might be attributed to its higher fluoride content, allowing for more enamel fluoride uptake, as fluoride ion was revealed to decline the enamel demineralization speed and improve enamel crystals development as well as more transformation of hydroxyapatite crystals into fluorapatite (27).

The null hypotheses investigated in the current study was rejected as the different tested remineralization protocols applied to white spot enamel lesions have different significant effect on enamel chemistry and microhardness.

## Conclusions

Under the limitation of this *in vitro* study; it can be concluded that hydroxyapatite crystals of the dental enamel can be successfully integrated with fluoride by the action of diode lasers along with the regular topical fluoride applications.

Further research is necessary upon the use of diode lasers regarding the enhancement of the tooth remineralization and micro-hardness.
